# Male Microchimerism in the Human Female Brain

**DOI:** 10.1371/journal.pone.0045592

**Published:** 2012-09-26

**Authors:** William F. N. Chan, Cécile Gurnot, Thomas J. Montine, Joshua A. Sonnen, Katherine A. Guthrie, J. Lee Nelson

**Affiliations:** 1 Clinical Research Division, Fred Hutchinson Cancer Research Center, Seattle, Washington, United States of America; 2 Department of Pathology, University of Washington, Seattle, Washington, United States of America; 3 Division of Rheumatology, University of Washington, Seattle, Washington, United States of America; Université de Montréal, Canada

## Abstract

In humans, naturally acquired microchimerism has been observed in many tissues and organs. Fetal microchimerism, however, has not been investigated in the human brain. Microchimerism of fetal as well as maternal origin has recently been reported in the mouse brain. In this study, we quantified male DNA in the human female brain as a marker for microchimerism of fetal origin (i.e. acquisition of male DNA by a woman while bearing a male fetus). Targeting the Y-chromosome-specific *DYS14* gene, we performed real-time quantitative PCR in autopsied brain from women without clinical or pathologic evidence of neurologic disease (n = 26), or women who had Alzheimer’s disease (n = 33). We report that 63% of the females (37 of 59) tested harbored male microchimerism in the brain. Male microchimerism was present in multiple brain regions. Results also suggested lower prevalence (p = 0.03) and concentration (p = 0.06) of male microchimerism in the brains of women with Alzheimer’s disease than the brains of women without neurologic disease. In conclusion, male microchimerism is frequent and widely distributed in the human female brain.

## Introduction

During pregnancy, genetic material and cells are bi-directionally exchanged between the fetus and mother [1], following which there can be persistence of the foreign cells and/or DNA in the recipient [2], [3]. This naturally acquired microchimerism (Mc) may impart beneficial or adverse effects on human health. Fetal Mc, which describes the persistence of cells and/or DNA of fetal origin in the mother acquired during pregnancy, has been associated with several different autoimmune diseases as well as implicated in tissue repair and immunosurveillance [4]–[6].

Although there is a broad anatomical distribution of Mc in humans that varies in prevalence and quantity [7]–[13], whether the human brain harbors fetal Mc and with what frequency is not known. Fetal Mc has recently been described in the mouse brain [14], [15]. In limited studies, maternal Mc was described in the human fetal brain [9].

In this study, we performed real-time quantitative PCR (qPCR) to detect and quantify male DNA in multiple brain regions of women, targeting the Y-chromosome-specific *DYS14* gene sequence as a marker for Mc of fetal origin. Deceased female subjects had no clinical or pathologic evidence of neurologic disease. We also tested brain specimens from women with Alzheimer’s disease (AD) for Mc. This is because AD has been reported as more prevalent in parous vs. nulliparous women [16], [17], increasing with higher number of pregnancies that also correlated with a younger age of AD onset [17], [18].

## Methods

### Subjects and Specimens

This study was approved by the institutional review board of the Fred Hutchinson Cancer Research Center (Number 5369; Protocol 1707). Subjects of the study were women without neurologic disease or with AD, totaling 59 deceased individuals. Twenty-six women had no neurologic disease. Thirty-three women had AD (Table 1). Brain autopsy specimens from these women came from one of two institutions: the Department of Pathology at the University of Washington in Seattle, Washington, or the Harvard Brain Tissue Resource Center established at McLean Hospital in Belmont, Massachusetts. Specimens from the University of Washington were obtained from adult women who had no clinical history of neurologic disease within two years of death and whose brain histology showed no evidence of disease, and from women who were diagnosed with probable AD during life [19] and met the National Institute on Aging-Reagan Institute consensus criteria for a neuropathologic diagnosis of AD [20]. Similarly, specimens from the Harvard Brain Tissue Resource Center were obtained from adult women without neurologic disease or who met clinical and pathologic criteria for AD. Age at death ranged from 32 to 101 (Table 1). Age at disease onset was known for subjects with AD from the University of Washington (median: 77 years; range: 64–93 years). Following autopsy, brain specimens were either formalin fixed or frozen in liquid nitrogen. Depending on availability, samples from two to twelve brain regions were obtained from each subject. Brain regions investigated included frontal lobe, parietal lobe, temporal lobe, occipital lobe, cingulate gyrus, hippocampus, amygdala, caudate, putamen, globus pallidus, thalamus, medulla, pons, cerebellum, and spinal cord. Subjects with AD contributed more specimens per person than subjects without neurologic disease, but this was not statistically significant (means: 3.6 vs. 2.5, respectively; p = 0.05). Combining subjects from both institutions, subjects with AD were significantly older at death (p<0.001); the post-mortem intervals (PMIs) were not significantly different (p = 0.06; Table 1). The most likely source of male Mc in female brain is a woman’s acquisition of male DNA from pregnancy with a male fetus. Limited pregnancy history was available on the subjects; pregnancy history on most subjects was unknown. Nine women were known to have at least one son, eight with AD and one without neurologic disease. Two women were known to have no history of having sons, one with AD and one without neurologic disease.

**Table 1:** Characteristics of female subjects without neurologic disease or with Alzheimer’s disease.

	No neurologic disease	Alzheimer’s disease
Number of subjects	26	33
Age at death: median (range)	70 (32–86) years	79 (54–101) years
PMI: median (range)	18.4 (4.0–30.6)	10.6 (2.0–28.6)
Braak stage: range (distribution)	None to II (None×6, I×11, II×7, Unknown×2)	III to VI (III×2, IV×2, V×9, VI×20)

### DNA Extraction

Genomic DNA was extracted from brain tissues using the QIAamp® DNA Mini Kit (QIAGEN, Valencia, CA) according to the manufacturer’s tissue protocol.

### Real-time qPCR

Male DNA was quantified in female brain tissues by amplifying the Y chromosome-specific sequence *DYS14* (GenBank Accession X06325) [21] using the TaqMan® assay and the ABI Prism® 7000 Sequence Detection System (Applied Biosystems, Foster City, CA). Primer and probe sequences for quantifying *DYS14* [22], as well as preparation of standard curves, composition of the qPCR mixture and thermal profile [23] have all been described previously. Square of the correlation coefficient for all standard curves was always greater than 0.99. Every experiment included no template controls to test for male DNA contamination during plate setup and all controls were negative. A minimum of six wells was tested for each specimen. Mean Ct was 36, with a range between 30 and 39 for all specimens except those of B6388, which was between 26 and 29. A representative amplification plot is provided in (Figure S1). Only wells in which amplification occurred at Ct<40 were used to calculate Mc concentration. In an individual well, the amount of DNA subject to qPCR did not exceed the equivalent of 3.5×10^4^ genomes (one genome is approximately equal to 6.6 pg of DNA), as this could inhibit amplification. The average amount of DNA in each well tested was determined by concurrent amplification of the beta globin sequence in separate, duplicate wells (primer and probe sequences described previously [24] except the reporter FAM was replaced with VIC). The final concentration of male Mc was expressed as the number of male genome equivalents (gEq) of DNA that would be detected in a specimen containing the equivalent of 1×10^5^ total genomes (gEq/10^5^). Total gEq tested per specimen did not differ between the two groups (medians: 1.3×10^5^ vs. 1.5×10^5^; p = 0.50). Because *DYS14* is a multi-copy gene [21] and our qPCR assay highly sensitive, and because of the possibility of cross contamination of specimens by male DNA of unknown origin before DNA extraction (i.e. during harvesting and handling of specimens) or from qPCR setup, we evaluated result positivity more conservatively by applying further criteria: 1) amplification occurred in at least two wells within a single experiment; and 2) total concentration of male Mc was >0.5 gEq/10^5^. Thus, estimates of male Mc might be lower than the true values. On the other hand, since detection of male DNA did not account for Mc potentially contributed by female fetuses, this could result in underestimation of the overall Mc in the brain. HLA-specific qPCR, as previously reported [25], [26], is another approach to Mc detection that is not sex-dependent. It requires participation of family members which was not possible for the current studies. As a supplementary study, we tested autopsied brain from a female systemic sclerosis patient by HLA-specific qPCR for whom we had familial HLA genotyping, targeting the child’s paternally transmitted HLA as previously described [26], [27]. These data are provided in (Tables S1 and S2). All qPCR data were analyzed using the 7000 System Sequence Detection Software.

### Statistics

Subject and Mc measurement characteristics were compared across groups by Chi-squared test for categorical data and t-test for continuous data. Mc prevalence and concentrations were analyzed according to disease status. A logistic regression model was used to estimate the association between Mc prevalence and disease status, with adjustment for total gEq tested, age at death, and PMI. The estimates were also adjusted for possible correlation between repeated measures from the same subject via generalized estimating equations. Association was reported as an odds ratio (OR) along with p value to indicate significance. As an example, OR of 0.30 could be interpreted to say that the odds of having AD for a subject who tested positive for Mc was 70% lower than the odds for a subject who tested negative. We also analyzed Mc concentrations as the outcome in Poisson log-linear regression models, assuming that the number of gEq detected as Mc was directly proportional to the number of total gEq tested. By definition, Mc occurs rarely, thus the data distribution is skewed to the right. We utilized negative-binomial models to account for the high degree of over-dispersion in the data; interpretation of the resulting estimates is identical to those of a Poisson model. Adjustments for potential confounders and for possible correlation between repeated measures were made as described above. The rate ratio (RR), along with p value to indicate significance, was used to compare the observed rates of Mc detection in the two groups. As an example, RR of 0.30 could be interpreted to say that the rate of Mc detection in subjects with AD was 70% lower than the rate of Mc detection in subjects without neurological disease. Secondary analysis was conducted to determine whether disease status was associated with Mc prevalence or concentration in a subset of samples from brain regions thought to be most affected by AD. Furthermore, we investigated whether Mc prevalence or concentration correlated with the Braak stage, which describes the extent of neurofibrillary tangles in subjects with AD [28], or with HLA-DRB1*1501, a human leukocyte antigen allele that has been reported in association with AD [29]. Two-sided p-values from regression models were derived from the Wald test. Analyses were performed on SAS software version 9 (SAS Institute, Inc., Cary, NC).

## Results

### Mc Prevalence and Concentration According to Brain Regions

**Figure 1:**
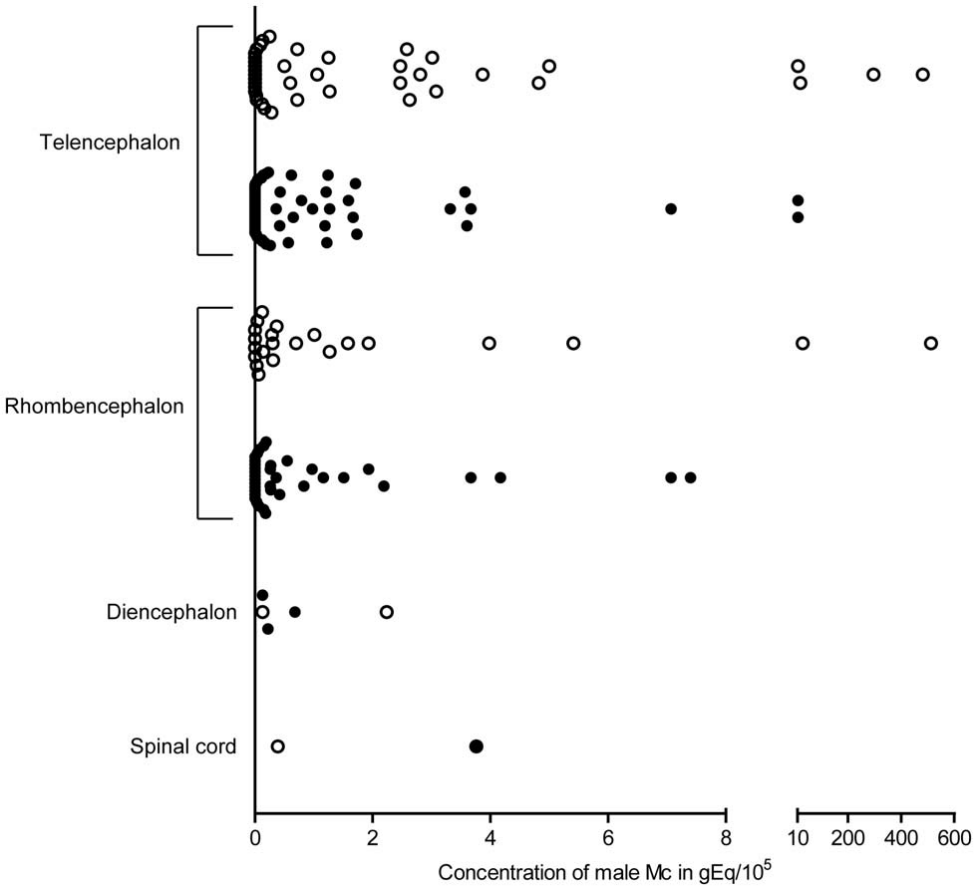
Concentration of male Mc in female human brain regions. Autopsied brain specimens of females without any neurologic disease (open circles) or with AD (filled circles) were tested by qPCR for male DNA. Each point represents one unique brain specimen. Telencephalon consists of neocortical regions (frontal, parietal, temporal, and occipital lobes), limbic regions (hippocampus, amygdala, and cingulate gyrus), and regions of the basal ganglia (putamen, caudate, and globus pallidus). Diencephalon consists of thalamus. Rhombencephalon consists of medulla, pons, and cerebellum. Due to the greater number of data points collected for telencephalon and rhombencephalon, data for each group have been plotted side by side to better present their distributions. Such separation was not done on the data for diencephalon and spinal cord.

The median number of specimens tested per subject was three, with a range of one to 12. Table 2 summarizes the specimen-level prevalence of male Mc according to brain region for all subjects. Per brain region, between two and 35 specimens were tested for male DNA. Although there were few specimens available, we did not detect male DNA in the frontal lobe and the putamen, and found the highest prevalence in the medulla. Considering all subjects together, Mc concentrations ranged from 0–512.5 gEq/10^5^, with a median value of 0.2 and a 90^th^ percentile of 3.7 gEq/10^5^ (Figure 1). One subject from the Harvard Brain Tissue Resource Center who was without neurologic disease (coded as B6388; age of death 69 years) had three specimens with the highest concentration values in our dataset (296.1, 481.8, and 512.5 gEq/10^5^ in the temporal lobe, cingulate gyrus, and pons, respectively). Using fluorescence *in situ* hybridization, we indeed found rare male cells in the brain of B6388 (Figure S2). The remaining concentration values in the dataset were 29.4 gEq/10^5^ or less. Regarding the relationship between pregnancy history and Mc prevalence, five of nine subjects who were known to have at least one son harbored male Mc in at least one of their brain regions (Table S3). All positive individuals had AD; among the negatives were three with AD and one without neurologic disease. One of two women without history of having sons was also positive for male Mc in her brain and without neurologic disease; the negative individual had AD.

**Table 2:** Prevalence of male Mc within individual brain regions in women without neurologic disease or with Alzheimer’s disease.

	Proportion of samples tested positive for male Mc (%)		
Brain region	Total	No neurologic disease	Alzheimer’s disease
**I. Telencephalon**			
i) *Neocortical*			
Frontal	0/3 (0)	–	0/3 (0)
Parietal	7/17 (41)	3/4 (75)	4/13 (31)
Temporal	12/26 (46)	6/13 (46)	6/13 (46)
Occipital	1/5 (20)	–	1/5 (20)
ii) *Limbic*			
Hippocampus	7/20 (35)	3/7 (43)	4/13 (31)
Amygdala	1/2 (50)	–	1/2 (50)
Cingulate gyrus	11/26 (42)	7/14 (50)	4/12 (33)
iii) *Basal ganglia*			
Putamen	0/4 (0)	–	0/4 (0)
Caudate	1/9 (11)	0/1 (0)	1/8 (13)
Globus pallidus	1/5 (20)	1/1 (100)	0/4 (0)
**II. Diencephalon**			
Thalamus	2/5 (40)	1/2 (50)	1/3 (33)
**III. Rhombencephalon**			
Medulla	7/8 (88)	1/1 (100)	6/7 (86)
Pons	11/35 (31)	7/17 (41)	4/18 (22)
Cerebellum	2/16 (13)	1/4 (25)	1/12 (8)
**IV. Spinal cord**	1/2 (50)	0/1 (0)	1/1 (100)
**All regions** *	64/183 (35)	30/65 (46)	34/118 (29)

* P = 0.03 comparing the overall prevalence of male Mc between the two groups. Individual brain regions were not compared due to limited sample sizes.

### Prevalence and Concentration of Male Mc in Human Brain: Women without Neurologic Disease or with AD

Of 183 specimens, 64 (35%) tested positive for Mc (Table 2). Eighteen of 26 subjects without neurologic disease (69%) had at least one positive value, with 30 positive results in 65 specimens (46%). Nineteen of 33 subjects with AD (58%) had at least one positive value, with 34 positive results in 118 specimens (29%). The estimated OR from a univariate model was 0.47 (95% confidence interval (CI) 0.21–1.08, p = 0.08). After adjustment for total gEq tested, age at death, and PMI, AD was significantly associated with lower Mc prevalence: OR 0.40 (95% CI 0.17–0.93, p = 0.03). Thus, the odds of having AD for a subject who tested positive for Mc was 60% lower than the odds for a subject who tested negative. When Mc concentrations were analyzed according to whether subjects had no neurologic disease or had AD, the estimated RR from an adjusted model was 0.05 (95% CI 0.01–0.39, p = 0.004). However, exclusion of brain specimens from subject B6388, who was without neurologic disease and whose level of male Mc was 10-fold greater than the next highest concentration from a different subject, changed the estimate dramatically: RR 0.41 (95% CI 0.16–1.05, p = 0.06). Thus, the rate of Mc detection in subjects with AD was 59% lower than the rate of Mc detection in subjects without neurological disease, but was not statistically significant. Age at death was also not statistically significantly associated with Mc prevalence, either in univariate or adjusted models (adjustments for disease status and total gEq tested; p = 0.79). However, any relationship between age at death and male Mc from prior pregnancies with a male fetus could not be evaluated because pregnancy history and the time interval from pregnancies to death were generally unknown from our subjects.

### Prevalence and Concentration of Male Mc in Brain Regions Affected by AD

We conducted a secondary analysis considering specimens only from the five brain regions thought to be most affected by AD: amygdala, hippocampus, frontal lobe, parietal lobe, and temporal lobe [30], [31]. Considering only these regions, 12 of 24 subjects without neurologic disease (50%) had at least one positive value, with 12 positive results in 24 specimens (50%). Thirteen of 33 subjects with AD (39%) had at least one positive value, with 15 positive results in 44 specimens (34%). The adjusted OR describing the association of Mc prevalence and disease status was 0.48 (95% CI 0.14–1.62, p = 0.23). Therefore, the odds of having AD for a subject who tested positive for Mc in brain regions most affected by this disease was 52% lower than the odds for a subject who tested negative, but was not statistically significant. However, none of the subjects without neurologic disease contributed specimens of the amygdala or the frontal lobe. Comparing Mc concentrations across groups, excluding one specimen from subject B6388, the adjusted RR was 0.27 (95% CI 0.13–0.56, p<0.001). Thus, the proportion of positive specimens was not significantly different between groups, but Mc concentrations in this subset of brain specimens from subjects with AD tended to have lower values than those found in subjects without neurologic disease. In other analyses, there was no significant association between Mc prevalence or concentration and the Braak stage (Table 1; p = 0.99 and 0.93, respectively), and no significant association between Mc prevalence and HLA-DRB1*1501 (8 of 11 DR15-bearing subjects positive for Mc (73%) vs. 16 of 31 subjects without DR15 who also had Mc (52%); p = 0.13).

## Discussion

In this study, we provide the first description of male Mc in female human brain and specific brain regions. Collectively with data showing the presence of male DNA in the cerebrospinal fluid [32], our results indicate that fetal DNA and likely cells can cross the human blood-brain barrier (BBB) and reside in the brain. Changes in BBB permeability occur during pregnancy [33] and may therefore provide a unique opportunity for the establishment of Mc in the brain. Also unique to our study are the findings that male Mc in the human female brain is relatively frequent (positive in 63% of subjects) and distributed in multiple brain regions, and is potentially persistent across the human lifespan (the oldest female in whom male DNA was detected in the brain was 94 years).

That Mc can penetrate the human BBB and reside in the brain was first indicated by murine studies that showed the presence of both foreign cells and DNA in mouse brains [14], [15]. However, prevalence of brain Mc in mice has not been well defined, as the frequency reported varies depending on the study [14], [15], [34]–[36], and in one investigation, Mc was not observed [37]. Similar to mouse data, our study of humans found that brain Mc was not present in all individuals tested. Even in those who showed positivity overall, not all of their brain regions had Mc. Mc concentration also showed considerable variability. Overall, our data complement and extend on other reports describing Mc in the general human population, in peripheral blood and at the level of the tissue/organ studied within and between subjects [9]–[13]. It is currently not possible to meaningfully compare Mc prevalence or concentration in human brain to other tissues because other tissues were not available from our subjects. Moreover, prior studies that evaluated Mc in other organs used diverse methods, some of which were not quantitative.

The most likely source of male Mc in female brain is acquisition of fetal Mc from pregnancy with a male fetus. In women without sons, male DNA can also be acquired from an abortion or a miscarriage [22], [23], [38]–[40]. The pregnancy history was unknown for all but a few subjects in the current studies, thus male Mc in female brain could not be evaluated according to specific prior pregnancy history. In addition to prior pregnancies, male Mc could be acquired by a female from a recognized or vanished male twin [41]–[43], an older male sibling, or through non-irradiated blood transfusion [44].

Because AD is more prevalent in women than men and an increased risk has been reported in parous vs. nulliparous women and correlated with younger age of onset [16]–[18], we also investigated male Mc in women with AD. AD is a neurodegenerative disease characterized by elevated levels of amyloid plaques, cerebrovascular amyloidosis, and neurofibrillary tangle [30]. Our results suggesting women with AD have a lower prevalence of male Mc in the brain and lower concentrations in regions most affected by AD were unexpected. However, the number of subjects tested was modest and, as discussed previously, pregnancy history was largely unknown. The explanation for decreased Mc in AD, should this observation be replicated in a larger study, is not obvious. In other diseases, both beneficial and detrimental effects of Mc of fetal origin have been described depending on several factors including the specific type and source of Mc [6]. A significant limitation of the current study was the inability to distinguish the type and source of male Mc, and further studies that distinguish genetically normal from abnormal Mc would be of potential interest.

At present, the biological significance of harboring Mc in the human brain requires further investigation. Mc appears to persist in the blood, bone, and bone marrow for decades [2], [45] and is present among different hematopoietic lineages [46]. Moreover, Mc appears to integrate and generate specific cell types in tissues [10], [11], [47]–[49]. In murine studies, fetal Mc in the maternal brain has been observed to resemble perivascular macrophages, neurons, astrocytes, and oligodendrocytes both morphologically and phenotypically and occupy the respective niches [15], [36]. Thus, it is possible that Mc in the brain is able to differentiate into various mature phenotypes or undergoes fusion with pre-existing cells and acquires a new phenotype, as suggested by murine and human studies in which bone marrow-derived cells circulated to the brain and generated neuronal cells by differentiation, or fused with pre-existing neurons [50]–[53]. Lastly, a few studies have reported an association between parity and decreased risk of brain cancer, raising the possibility that Mc could contribute to immunosurveillance against tumorigenic cells as has been suggested for some other types of malignancy [6], [54]–[56].

In conclusion, male Mc is frequent and widely distributed in the human female brain. Although the relationship between brain Mc and health versus disease requires further study, our findings suggest that Mc of fetal origin could impact maternal health and potentially be of evolutionary significance.

## Supporting Information

Figure S1 ***DYS14*** **amplification plot of a representative brain specimen.** Shown is the amplification plot of a sample of the cingulate from a female with AD.
(TIF)

Figure S2 **Detection of male nuclei in female brain by fluorescence** ***in situ*** **hybridization.** Formalin-fixed, paraffin-embedded pons of subject B6388, in whom male Mc was quantified at a concentration of 512.5 gEq/10^5^ by qPCR, was hybridized with fluorescent probes specific for X and Y chromosomes. Tissue section was deparaffinized, rehydrated, denatured in a solution of 44% formic acid and 0.3% hydrogen peroxide for 15 minutes, and incubated in antigen retrieval solution (Dako, Carpinteria, CA) in a conventional food steamer for more than 30 minutes. Further denaturation was done using 0.2 N hydrochloric acid for 20 minutes. Section was washed with 2×sodium chloride/sodium citrate (SSC) containing 0.05% Tween-20, incubated in 2×SSC at 42°C for 30 minutes, and treated with pepsin (DIGEST-ALL™ 3, Dako) at 37°C for 10 minutes. Section was dehydrated, air-dried, and fluorescent probes specific for X and Y chromosomes were diluted in a hybridization mixture (10% dextran sulfate, 2×SSC, and 50% formamide) and applied using a cover slip. X and Y chromosome probes have been described previously (Lau et al. Lancet 1∶14–16; Waye and Willard. Nucleic Acids Res 13∶2731–2743) and were conjugated to Alexa Fluor 555 and 488, respectively, using the FISH Tag™ DNA Kit (Molecular Probes, Eugene, OR). Probes were denatured at 90°C for 10 minutes. Hybridization was done overnight in a humidified chamber at 37°C for 16 hours. After hybridization, section was washed in 2×SSC, followed by two washes in 2×SSC containing 50% formamide, and a final wash in 2×SSC. All post-hybridization washes were done at 42°C for 5 minutes. Section was treated with a 50 mM ammonium acetate buffer (pH 5) containing 1 mM cupric sulfate for 30 minutes to reduce autofluorescence, then mounted with VECTASHIELD® Mounting Medium containing 4′,6-diamidino-2-phenylindole (Vector Laboratories, Burlingame, CA). To facilitate detection of rare male nuclei in a large background of female nuclei, section was visualized by automated scanning, using a TissueFAXS System (TissueGnostics USA, Tarzana, CA) in fluorescence mode and the 40×objective. Images were manually reviewed for any nucleus simultaneously expressing red and green signals. Putative male nuclei were re-examined using the 100×objective under oil immersion, and images were collected with the Z-stack option selected. Images were deconvolved using Volocity® 3D Image Analysis Software (PerkinElmer, Waltham, MA). An example of a male nucleus is shown (indicated by arrow and re-shown in white box) at a final magnification of 400×. Hybridization signals were confirmed to be nuclear in location following deconvolution of the Z-stack images containing the putative male nucleus (not shown).
(TIF)

Table S1 **HLA genotyping of a family for investigating fetal origin microchimerism in the mother’s brain.**
(DOC)

Table S2 **Quantification of fetal origin microchimerism in the mother’s brain by HLA-specific qPCR.**
(DOC)

Table S3 **Mc prevalence and concentration in women known to have at least one son.**
(DOCX)
