# Supplementary figures and images for: Male Microchimerism in the Human Female Brain
Source: PLoS One. 2012 Sep 26;7(9):e45592. doi: 10.1371/journal.pone.0045592 (PMC3458919; doi:10.1371/journal.pone.0045592)

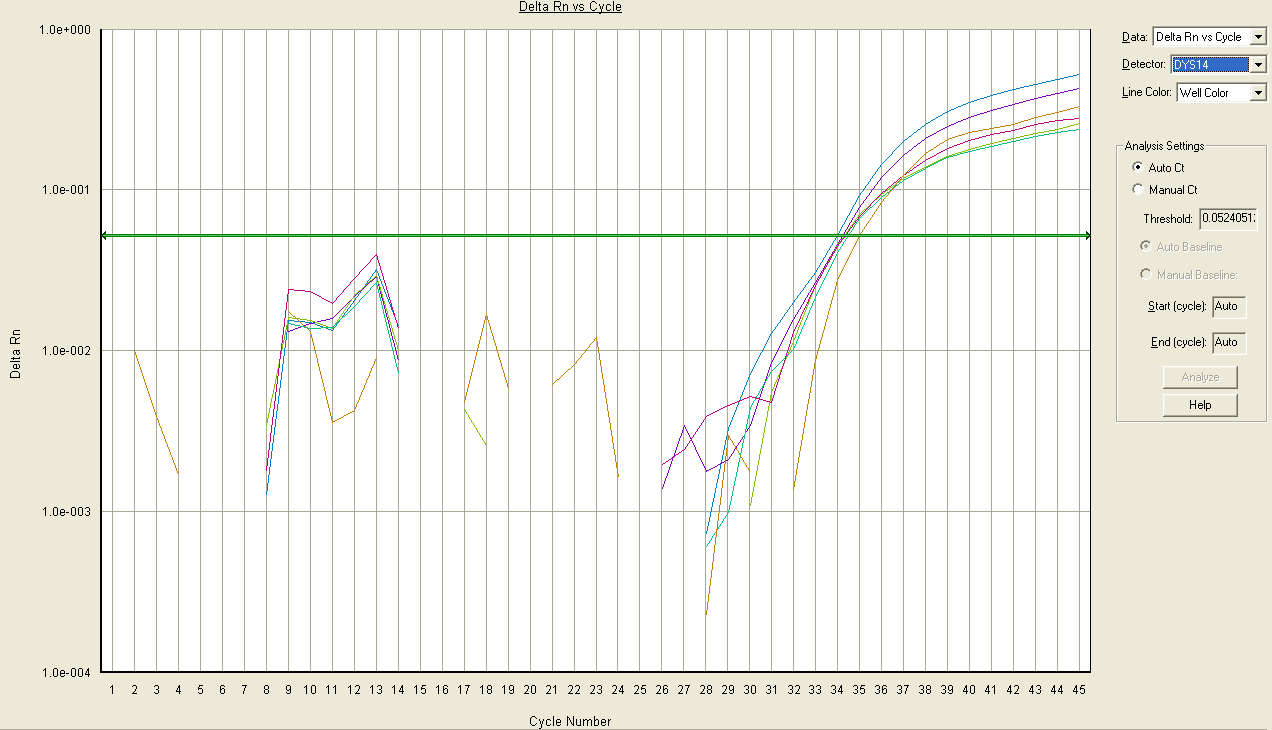

Supplement: Figure S1 — DYS14 amplification plot of a representative brain specimen. Shown is the amplification plot of a sample of the cingulate from a female with AD. (TIF) [file pone.0045592.s001.tif]

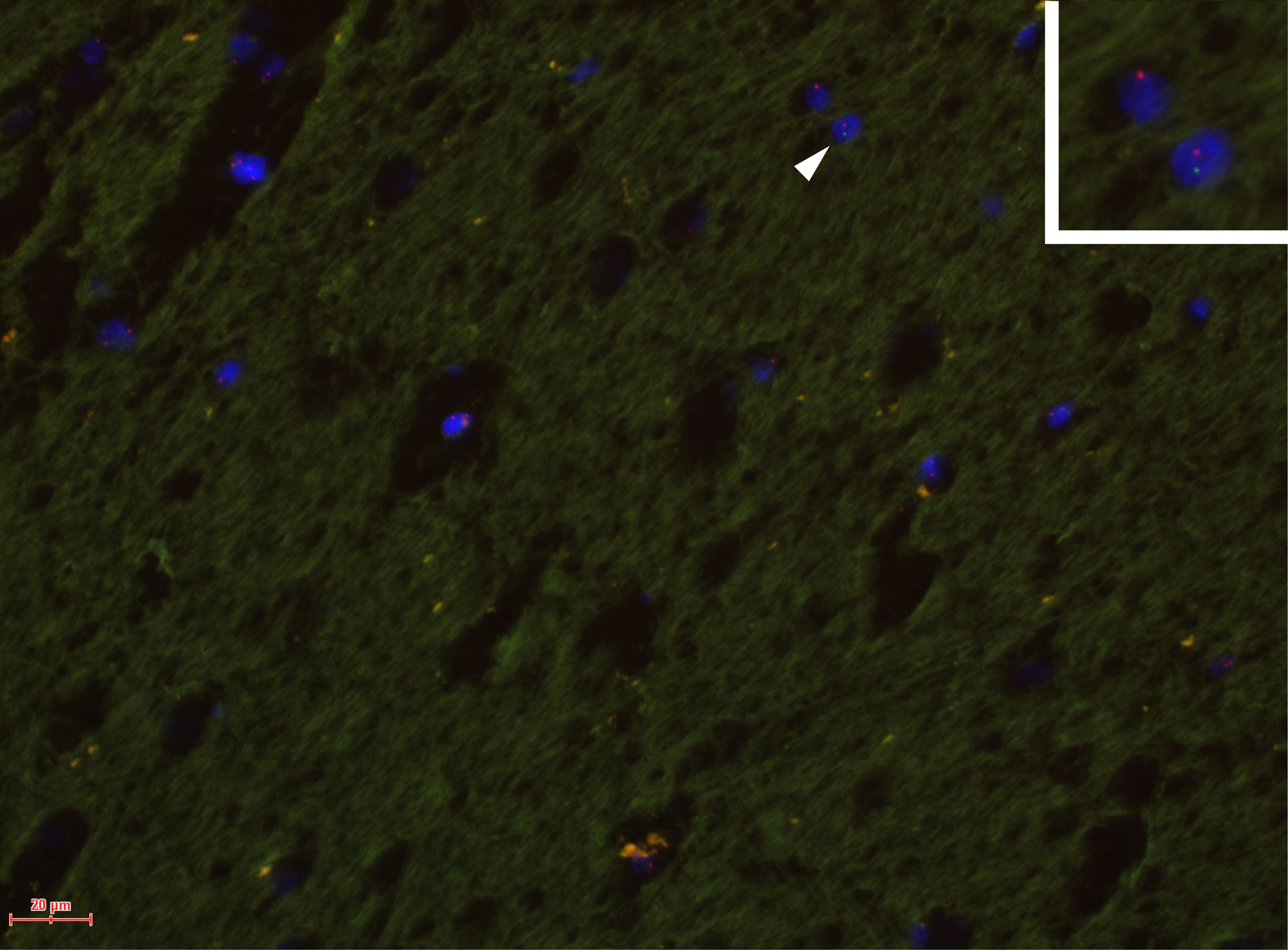

Supplement: Figure S2 — Detection of male nuclei in female brain by fluorescence in situ hybridization. Formalin-fixed, paraffin-embedded pons of subject B6388, in whom male Mc was quantified at a concentration of 512.5 gEq/105 by qPCR, was hybridized with fluorescent probes specific for X and Y chromosomes. Tissue section was deparaffinized, rehydrated, denatured in a solution of 44% formic acid and 0.3% hydrogen peroxide for 15 minutes, and incubated in antigen retrieval solution (Dako, Carpinteria, CA) in a conventional food steamer for more than 30 minutes. Further denaturation was done using 0.2 N hydrochloric acid for 20 minutes. Section was washed with 2×sodium chloride/sodium citrate (SSC) containing 0.05% Tween-20, incubated in 2×SSC at 42°C for 30 minutes, and treated with pepsin (DIGEST-ALL™ 3, Dako) at 37°C for 10 minutes. Section was dehydrated, air-dried, and fluorescent probes specific for X and Y chromosomes were diluted in a hybridization mixture (10% dextran sulfate, 2×SSC, and 50% formamide) and applied using a cover slip. X and Y chromosome probes have been described previously (Lau et al. Lancet 1∶14–16; Waye and Willard. Nucleic Acids Res 13∶2731–2743) and were conjugated to Alexa Fluor 555 and 488, respectively, using the FISH Tag™ DNA Kit (Molecular Probes, Eugene, OR). Probes were denatured at 90°C for 10 minutes. Hybridization was done overnight in a humidified chamber at 37°C for 16 hours. After hybridization, section was washed in 2×SSC, followed by two washes in 2×SSC containing 50% formamide, and a final wash in 2×SSC. All post-hybridization washes were done at 42°C for 5 minutes. Section was treated with a 50 mM ammonium acetate buffer (pH 5) containing 1 mM cupric sulfate for 30 minutes to reduce autofluorescence, then mounted with VECTASHIELD® Mounting Medium containing 4′,6-diamidino-2-phenylindole (Vector Laboratories, Burlingame, CA). To facilitate detection of rare male nuclei in a large background of female nuclei, section was visualized by automated s [file pone.0045592.s002.tif]
