# Supplementary material for: Male Microchimerism in the Human Female Brain
Source: PLoS One. 2012 Sep 26;7(9):e45592. doi: 10.1371/journal.pone.0045592 (PMC3458919; doi:10.1371/journal.pone.0045592)
Supplement: Table S1 — HLA genotyping of a family for investigating fetal origin microchimerism in the mother’s brain. (DOC) [file pone.0045592.s003.doc]

Table S1. HLA genotyping* of a family for investigating fetal origin microchimerism in the mother’s brain.

| **Subject** | **DRB1(i)**^&^ | **DRB1(ii)**^&^ | **DQA1(i)**^&^ | **DQA1(ii)**^&^ | **DQB1(i)**^&^ | **DQB1(ii)**^&^ |
| --- | --- | --- | --- | --- | --- | --- |
| 179A^‡^ | 1303 | 0804 | 0505 | 0505 | 0301 | 0301 |
| 179B^‡^ | 0402 | 0404 | 0301 | 0301 | 0302 | 0302 |
| 179C6^‡^ | 1303 | 0404^¶^ | 0505 | 0301^¶^ | 0301 | 0302 |

^*^ HLA-DRB1, DQA1 and DQB1 genotyping was performed on the indicated subjects using the LABType® SSO DNA typing system and a LABScan™ 100 flow analyzer (One Lambda, Canoga Park, CA), according to manufacturer’s instructions. Alleles were assigned using the HLA Fusion™ software.

^&^ (i) and (ii) denote the two haplotypes.

^‡^ The mother is designated “A”. Her spouse is designated “B”. “C6” denotes the sixth child born to them out of a total of six children. The woman who was studied had systemic sclerosis. Her child had Down syndrome.

^¶^ Informative HLA sequences (from the mother’s perspective) are highlighted in gray. Although the child’s DQB1*0302 also differs from the mother, we do not have an HLA-specific qPCR assay that can distinguish DQB1*0302 in a DQB1*0301 background.
