# Supplementary material for: Male Microchimerism in the Human Female Brain
Source: PLoS One. 2012 Sep 26;7(9):e45592. doi: 10.1371/journal.pone.0045592 (PMC3458919; doi:10.1371/journal.pone.0045592)
Supplement: Table S2 — Quantification of fetal origin microchimerism in the mother’s brain by HLA-specific qPCR. (DOC) [file pone.0045592.s004.doc]

Table S2. Quantification of fetal origin microchimerism in the mother’s brain by HLA-specific qPCR*.

| **HLA sequence tested** | **Source of Mc** | **Total gEq tested** | **Mc in gEq/10^5^** |
| --- | --- | --- | --- |
| DRB1*04 | Fetal | 229,556 | 2.60 |
| DQA1*03 | Fetal | 156,268 | 0.99 |

^*^ HLA-specific qPCR was performed according to our previously described method (Lambert NC et al. (2004) Arthritis Rheum 50: 906-914). All assays were run using the ABI Prism® 7000 Sequence Detection System (Applied Biosystems, Foster City, CA). Data were analyzed using the 7000 System Sequence Detection Software. The concentration of Mc was expressed per 100,000 gEq.
