# Supplementary material for: Male Microchimerism in the Human Female Brain
Source: PLoS One. 2012 Sep 26;7(9):e45592. doi: 10.1371/journal.pone.0045592 (PMC3458919; doi:10.1371/journal.pone.0045592)
Supplement: Table S3 — Mc prevalence and concentration in women known to have at least one son. (DOCX) [file pone.0045592.s005.docx]

Table S3. Mc prevalence and concentration in women known to have at least one son.

| **Subject** | **Number of sons** | **Number of brain regions positive for male DNA** | **Number of brain regions tested** | **Mc concentration of positive region(s) (gEq/10^5^)** |
| --- | --- | --- | --- | --- |
| 1 | 1 | 0 | 3 | NA^&^ |
| 2 | 1 | 0 | 10 | NA |
| 3 | 2 | 0 | 6 | NA |
| 4 | 2 | 1 | 3 | 1.2 |
| 5 | 2 | 1 | 3 | 17.1 |
| 6 | 2 | 3 | 3 | 0.9, 2.0, 9.7 |
| 7 | 4 | 1 | 3 | 4.4 |
| 8^†^ | ≥1^*^ | 0 | 3 | NA |
| 9 | ≥1^*^ | 2 | 3 | 0.6, 7.5 |

^&^ Not applicable.

^†^ This subject did not have a history of neurologic disease. All remaining subjects had AD.

^*^ Subjects 8 and 9 were known to have sons but the exact numbers were unknown.
